# Renal Challenges in Pregnancy: Clinical Spectrum and Outcomes of Acute Kidney Injury in a Tertiary Care Center

**DOI:** 10.7759/cureus.104357

**Published:** 2026-02-27

**Authors:** Rajni Agrawal, Aarti G Suryawanshi, Kanupriya Verma, Archana Bharti, Deepshikha Verma, Sharmila

**Affiliations:** 1 Obstetrics and Gynaecology, Maharaja Agrasen Medical College, Hisar, IND; 2 Anesthesiology, Maharaja Agrasen Medical College, Hisar, IND

**Keywords:** dialysis, maternal outcome, perinatal mortality, pregnancy-related acute kidney injury, sepsis

## Abstract

Background

Pregnancy-related acute kidney injury (PR-AKI) remains a major contributor to maternal and perinatal morbidity and mortality, particularly in low- and middle-income countries such as India. While the incidence of PR-AKI has declined globally, preventable causes such as preeclampsia, sepsis, and hemorrhage continue to predominate in resource-limited settings. This study aimed to evaluate the clinical profile, etiological spectrum, maternal and perinatal outcomes, and predictors of adverse prognosis among women with PR-AKI.

Methods

A prospective observational study was conducted at a tertiary care teaching hospital in India, including 120 women diagnosed with PR-AKI over a one-year period. Baseline demographics, antenatal care utilization, etiologies, laboratory parameters, and management interventions (including dialysis and ICU admission) were recorded. Maternal outcomes (mortality, dialysis dependence, and recovery) and perinatal outcomes (gestational age at delivery, low birth weight, neonatal intensive care unit (NICU) admission, and perinatal mortality) were analyzed. Statistical comparisons were made using chi-square and t-tests, with logistic regression applied to identify independent predictors of adverse outcomes.

Results

The mean maternal age was 26.9 ± 4.8 years, with 61.7% residing in rural areas and 45% lacking formal antenatal care (ANC) registration. The leading etiologies were preeclampsia/eclampsia (36.7%), sepsis (28.3%), and hemorrhage (19.2%). Dialysis was required in 42 (35%) women, and 18 (15%) required ICU admission. Maternal mortality was 14.2%, while 11% remained dialysis-dependent at discharge. Perinatal outcomes were poor: mean gestational age was 35.4 ± 3.8 weeks, 56.3% of neonates were low birth weight, 34% required NICU admission, and perinatal mortality reached 32.1%. Independent predictors of adverse maternal outcomes included delayed hospital presentation (>48 hours), KDIGO (kidney disease: improving global outcomes) stage 3, dialysis requirement, and ICU admission, while adverse perinatal outcomes were associated with unregistered ANC status, severe renal dysfunction (creatinine > 5 mg/dL), thrombocytopenia, and preterm delivery.

Conclusion

PR-AKI continues to exert a substantial burden in India, with high maternal and perinatal morbidity and mortality. Timely recognition, adequate antenatal care coverage, and early referral remain crucial in improving outcomes. Preventive strategies targeting sepsis, hypertensive disorders, and hemorrhage, alongside strengthening dialysis and critical care access, are essential for reducing the dual burden of maternal and perinatal loss.

## Introduction

Pregnancy is accompanied by profound physiological adaptations in renal function. Renal plasma flow and glomerular filtration rate increase by almost 50% during early gestation, facilitating maternal metabolic clearance and maintaining fetal growth requirements [1]. While these changes are usually beneficial, they render the kidneys susceptible to decompensation when additional stressors, such as hypertensive disorders, hemorrhage, or infections, arise [2].

Pregnancy-related acute kidney injury (PR-AKI) is a serious complication that can occur at any stage of gestation or in the postpartum period. Although its incidence has declined in developed countries to <1 per 20,000 pregnancies, largely due to safer obstetric practices, it continues to represent a significant problem in low- and middle-income countries [3,4]. In South Asia, PR-AKI contributes to 5%-15% of all acute kidney injury cases admitted to tertiary hospitals [5], with maternal mortality rates ranging from 5% to 20% and perinatal mortality often exceeding 30% [6,7].

The etiology of PR-AKI varies regionally. In high-income countries, it is most often associated with preeclampsia, HELLP (hemolysis, elevated liver enzymes, and low platelets) syndrome, and preexisting renal disease [8]. By contrast, in developing nations, preventable causes, such as puerperal sepsis, unsafe abortions, obstructed labor, and obstetric hemorrhage, remain dominant [9]. Notably, hypertensive disorders of pregnancy alone account for nearly 40% of cases worldwide, while sepsis and hemorrhage together contribute to another 30%-40% [4,10].

Beyond maternal health, PR-AKI has profound consequences for perinatal well-being. Impaired renal function and hemodynamic instability compromise uteroplacental circulation, predisposing to intrauterine growth restriction, preterm birth, stillbirth, and increased neonatal intensive care admissions [6,11]. Even in cases where maternal renal function recovers, neonates are at higher risk of low birth weight and perinatal mortality compared with unaffected pregnancies [7].

Despite advances in critical care and dialysis support, a proportion of women progress to end-stage renal disease (ESRD) or remain dialysis-dependent after pregnancy [5,9]. Furthermore, PR-AKI is now recognized as a risk factor for long-term chronic kidney disease and cardiovascular complications in survivors [2,8]. These sequelae highlight the need for early recognition, preventive strategies, and comprehensive follow-up care.

However, data from India and other resource-limited settings remain scarce, fragmented, and often hospital-specific. This lack of population-based evidence limits the development of effective public health policies and clinical protocols. Against this backdrop, the present study aimed to describe the clinical profile, etiological spectrum, and maternal and perinatal outcomes among women diagnosed with pregnancy-related acute kidney injury (PR-AKI) admitted to a tertiary care center.

## Materials and methods

Study design and setting

This was a prospective observational study conducted in the Department of Obstetrics and Gynecology in collaboration with the Department of Nephrology at a tertiary care teaching hospital in North India. The study was carried out over a 12-month period from January 2022 to December 2022. The institution functions as a major referral center catering to both rural and urban populations. Ethical approval was obtained from the Institutional Ethics Committee (Approval no.: MAMC/2021/IEC/232). Written informed consent was obtained from all participants or their legally authorized representatives.

Study population and sample size

The study included all pregnant women and postpartum women up to six weeks after delivery who were admitted with a diagnosis of acute kidney injury during the study period. Acute kidney injury was defined according to the KDIGO (kidney disease: improving global outcomes) criteria [12]. Women with known chronic kidney disease, prior renal transplantation, or those on maintenance dialysis before pregnancy were excluded to ensure inclusion of pregnancy-specific acute renal insults only. Women unwilling to participate were also excluded. All consecutive eligible patients presenting during the study period were enrolled, constituting a convenience sample. A total of 120 women met the inclusion criteria and were included in the final analysis.

Data collection and clinical evaluation

Data were collected prospectively using a predesigned structured proforma. Baseline variables included maternal age, parity, antenatal care registration, socioeconomic status, gestational age at presentation, referral status, time to hospital presentation, and associated comorbidities. Clinical parameters such as blood pressure, urine output, and signs of fluid imbalance were recorded. Laboratory investigations included hemoglobin, platelet count, serum creatinine, blood urea nitrogen, serum electrolytes, liver function tests, coagulation profile, lactate levels, and urine analysis, including protein estimation. Renal ultrasonography was performed in all patients to assess kidney size and exclude obstructive causes. Microbiological cultures were obtained where clinically indicated.

Outcome measures

The primary maternal outcomes studied were the requirement of renal replacement therapy, intensive care unit admission, renal recovery status at discharge, and maternal mortality. Renal recovery was categorized as complete, partial, or dialysis-dependent. The primary perinatal outcomes included gestational age at delivery, preterm birth, birth weight, Apgar scores, NICU admission, stillbirth, early neonatal death, and perinatal mortality. Secondary outcomes included identification of clinical and laboratory predictors associated with adverse maternal and perinatal outcomes.

Statistical analysis

Data were coded and analyzed using IBM SPSS Statistics, version 25.0 (IBM Corp., Armonk, NY). Normality was assessed using the Shapiro-Wilk test. Continuous variables were summarized as mean ± SD or median (IQR) as appropriate and categorical variables as frequencies and percentages (n (%)). Associations between categorical variables were analyzed using the chi-square test or Fisher’s exact test, while continuous variables were compared using independent t-tests or Mann-Whitney U tests based on data distribution. Variables with p < 0.10 on univariate analysis were entered into multivariable binary logistic regression models to identify independent predictors of adverse maternal and perinatal outcomes. Results were expressed as adjusted odds ratios (aOR) with 95% confidence intervals, and Wald χ² statistics were reported. All tests were two-tailed, and p < 0.05 was considered statistically significant.

## Results

Among 120 women with PR-AKI, the mean age was 27.8 ± 4.6 years, with 82 (68.3%) aged <30 years. A majority, 78 (65.0%), belonged to low socioeconomic status, 89 (74.2%) resided in rural areas, and 50 (41.7%) had no antenatal visits, reflecting inadequate antenatal care. Primigravidae constituted 72 (60.0%) patients. Comorbidities were identified in 34 (28.3%) patients, most commonly chronic hypertension in 16 (13.3%) and severe anemia in 26 (21.7%). The mean gestational age at AKI diagnosis was 33.6 ± 10.7 weeks, with half presenting in the third trimester. Notably, 84 (70.0%) were referrals from peripheral facilities, and 46 (38.3%) presented >48 hours after symptom onset (Table 1).

**Table 1:** Baseline demographic and clinical characteristics of women with pregnancy-related acute kidney injury (n = 120) ANC: Antenatal care; NSAID: Non-steroidal anti-inflammatory drugs; AKI: Acute kidney injury.

Variables	Frequency (%)/Mean ± SD
Age (years)	27.8 ± 4.6
*Age group*	
<30 years	82 (68.3)
≥30 years	38 (31.7)
BMI (kg/m²)	22.6 ± 3.1
*Education*	
≤10th standard	72 (60.0)
>10th standard	48 (40.0)
*Socioeconomic status*	
Low	78 (65.0)
Middle	36 (30.0)
High	6 (5.0)
*Residence*	
Rural	89 (74.2)
Urban	31 (25.8)
*Registration for ANC*	
No	45 (37.5)
Yes	75 (62.5)
*Antenatal visits*	
None	50 (41.7)
1–3	32 (26.7)
≥4	38 (31.7)
*Parity*	
Primigravida	72 (60.0)
Multigravida	48 (40.0)
Multiple gestation	8 (6.7)
Consanguinity	10 (8.3)
Prior hypertensive disorder (previous pregnancy)	14 (11.7)
Comorbidities	34 (28.3)
Chronic hypertension	16 (13.3)
Diabetes	8 (6.7)
Hypothyroid	10 (8.3)
Severe anemia (Hb < 7 g/dL)	26 (21.7)
Baseline creatinine available pre-pregnancy	22 (18.3)
Baseline creatinine available pre-pregnancy (mg/dL)	0.6 ± 0.1
Substance/NSAID use in current pregnancy	9 (7.5)
Gestational age at AKI diagnosis (weeks)	33.6 ± 10.7
*Trimester at presentation*	
1st	10 (8.3)
2nd	28 (23.3)
3rd	60 (50.0)
Postpartum	22 (18.4)
Referred from the peripheral facility	84 (70.0)
Distance to hospital > 50 km	52 (43.3)
Time from symptom onset to hospital >48 hours	46 (38.3)
Time from symptom onset to hospital	30.3 ± 14.6

Among the 120 women with PR-AKI, 98 were diagnosed during pregnancy and 22 during the postpartum period. Overall, hypertensive disorders were the most frequently observed etiological category, identified in 45 women (37.5%), predominantly preeclampsia in 28 (23.3%), followed by eclampsia in nine (7.5%) and HELLP syndrome in eight (6.7%). Obstetric hemorrhage was noted in 24 (20.0%) patients, comprising antepartum hemorrhage in seven (5.8%) and postpartum hemorrhage in 17 (14.2%). Sepsis accounted for 28 (23.3%) cases, including puerperal sepsis or endometritis in 12 (10.0%) and urinary tract infection or acute pyelonephritis in 10 (8.3%). Less frequent etiologies included septic abortion in 10 (8.3%) patients, obstructed or prolonged labor in six (5.0%), and other medical causes in seven (5.8%), such as acute fatty liver of pregnancy in three (2.5%), viral hepatitis E in two (1.7%), malaria in one (0.8%), and dengue in one (0.8%). Thrombotic microangiopathies and iatrogenic or drug-related causes were observed in three (2.5%) cases each. Multiple concurrent etiologies were identified in 19 women (15.8%). Given that the majority of cases occurred during pregnancy, postpartum-specific causes such as puerperal sepsis and postpartum hemorrhage should be interpreted cautiously, as their relative frequencies reflect the smaller postpartum representation in the study cohort rather than a lower etiological importance (Table 2).

**Table 2:** Etiological spectrum of pregnancy-related acute kidney injury (n = 120) HELLP: Hemolysis, elevated liver enzymes, and low platelets; TTP: Thrombotic thrombocytopenic purpura; aHUS: Atypical hemolytic uremic syndrome.

Etiology	Total	During Pregnancy (n = 98)	Postpartum (n = 22)
	Frequency (%)		
Hypertensive disorders	45 (37.5)	45 (45.9)	0 (0.0)
─ Preeclampsia	28 (23.3)	28 (28.6)	0 (0.0)
─ Eclampsia	9 (7.5)	9 (9.2)	0 (0.0)
─ HELLP syndrome	8 (6.7)	8 (8.2)	0 (0.0)
Obstetric hemorrhage (total)	24 (20.0)	7 (7.1)	17 (77.3)
─ Antepartum hemorrhage	7 (5.8)	7 (7.1)	0 (0.0)
─ Postpartum hemorrhage	17 (14.2)	0 (0.0)	17 (77.3)
Sepsis (non-abortion)	28 (23.3)	16 (16.3)	12 (54.5)
─ Puerperal sepsis/Endometritis	12 (10.0)	0 (0.0)	12 (54.5)
─ UTI acute pyelonephritis	10 (8.3)	10 (10.2)	0 (0.0)
─ Other infections	6 (5.0)	6 (6.1)	0 (0.0)
Septic abortion	10 (8.3)	10 (10.2)	0 (0.0)
Obstructed/Prolonged labor	6 (5.0)	6 (6.1)	0 (0.0)
Volume depletion	7 (5.9)	7 (7.1)	0 (0.0)
Other medical causes	7 (5.8)	7 (7.1)	0 (0.0)
─ Acute fatty liver of pregnancy	3 (2.5)	3 (3.1)	0 (0.0)
─ Viral hepatitis E	2 (1.7)	2 (2.0)	0 (0.0)
─ Malaria	1 (0.8)	1 (1.0)	0 (0.0)
─ Dengue	1 (0.8)	1 (1.0)	0 (0.0)
Thrombotic microangiopathy (TTP/aHUS)	3 (2.5)	3 (3.1)	0 (0.0)
Iatrogenic/Drug-related	3 (2.5)	2 (2.0)	1 (4.5)
≥2 concurrent etiologies	19 (15.8)	15 (15.3)	4 (18.2)

At presentation, mean blood pressure was elevated (154.6/96.4 mmHg). Oliguria occurred in 81 (67.5%) and anuria in 22 (18.3%) patients. The mean serum creatinine was 5.1 ± 2.3 mg/dL, with a median peak of 6.2 mg/dL (IQR: 4.6-8.1); 67 (55.8%) were classified as KDIGO stage 3. Hematologic abnormalities were frequent, with anemia (mean hemoglobin 8.4 g/dL) and thrombocytopenia in 34 (28.3%). Biochemical derangements included hyperkalemia (mean: 5.6 mEq/L), elevated lactate dehydrogenase (LDH) (mean: 712.6 U/L), and proteinuria in 66 (55.0%) women. Renal ultrasound revealed normal-sized kidneys in 112 (93.3%), with increased echogenicity in 18 (15.0%) and hydronephrosis in six (5.0%) patients. Blood or urine cultures were positive in 26 (21.6%), predominantly *Escherichia coli* in 12 (10.0%) patients (Table 3).

**Table 3:** Clinical and laboratory characteristics of women with pregnancy-related acute kidney injury (n = 120) AST: Aspartate transaminase; ALT: Alanine transaminase; LDH: Lactate dehydrogenase; UPCR: Urine protein–creatinine ratio; INR: International normalized ratio; aPTT: Activated partial thromboplastin time; KDIGO: Kidney disease: improving global outcomes.

Parameters	Frequency (%)/Mean ± SD/Median (IQR)
Systolic/Diastolic BP (mmHg)	154.6 ± 21.7/96.4 ± 14.2
Oliguria (<400 mL/24 h)	81 (67.5)
Anuria	22 (18.3)
Serum creatinine (mg/dL)	5.1 ± 2.3
Peak creatinine during admission (mg/dL)	6.2 (4.6–8.1)
Blood urea nitrogen (mg/dL)	92.5 ± 36.4
Serum potassium (mEq/L)	5.6 ± 1.2
Hemoglobin (g/dL)	8.4 ± 1.9
Platelet count (×10⁹/L)	128 (82–176)
Thrombocytopenia <100 × 10⁹/L	34 (28.3)
AST (U/L)	78.4 ± 52.1
ALT (U/L)	64.7 ± 41.5
LDH (U/L)	712.6 ± 236.5
Total bilirubin (mg/dL)	1.6 ± 0.9
Serum uric acid (mg/dL)	7.4 ± 1.8
Urine protein ≥2+ on dipstick	52 (43.3)
Spot urine protein–creatinine ratio	0.9 (0.4–2.2)
Proteinuria (UPCR ≥ 0.3 g/g or ≥+1 dipstick)	66 (55.0)
Total leukocyte count (×10⁹/L), mean ± SD	13.4 ± 5.2
Serum lactate (mmol/L), mean ± SD	2.6 ± 1.3
Coagulopathy (INR ≥ 1.5 or aPTT > 1.5×)	18 (15.0)
KDIGO stage at peak	
Stage 1	22 (18.3)
Stage 2	31 (25.8)
Stage 3	67 (55.8)
Renal ultrasound	
Normal size	112 (93.3)
Increased echogenicity	18 (15.0)
Hydronephrosis	6 (5.0)
Positive cultures (blood/urine)	26 (21.6)
Escherichia coli	12 (10.0)
Klebsiella	6 (5.0)
Staphylococcus aureus	4 (3.3)
Others	4 (3.3)

ICU admission was required in 32 (26.7%) patients, and 55 (45.8%) underwent renal replacement therapy, primarily intermittent hemodialysis. Blood product transfusion was administered to 58 (48.3%), while vasopressors and mechanical ventilation were required in 24 (20.0%) and 19 (15.8%) patients, respectively. Dialysis was initiated within 24 hours in most cases, though 19 (34.5%) experienced dialysis-related complications, mainly intradialytic hypotension in 16 (29.1%) patients. Obstetric complications included postpartum hemorrhage in 19 (15.8%) and placental abruption in six (5.0%) patients. Cesarean section accounted for 42 (37.5%) deliveries, while pregnancy termination occurred in eight (6.6%) patients. At discharge, 65 (54.2%) achieved complete renal recovery, 18 (15.0%) partial recovery, and 10 (8.3%) remained dialysis-dependent. Maternal mortality was 25 (20.8%), primarily due to sepsis in 12 (48.0%). Median ICU and hospital stays were four (IQR 2-8) and nine (IQR 6-14) days, respectively. On follow-up, 72 (75.7%) of 95 survivors maintained eGFR ≥ 60 mL/min/1.73 m², while 10 (10.6%) remained dialysis-dependent (Table 4).

**Table 4:** Interventions, complications, and outcomes among women with pregnancy-related acute kidney injury (n = 120; 112 deliveries) *Among those who received the RRT. HDP: Hypertensive disorders in pregnancy; RRT: Renal replacement therapy; PPH: Postpartum hemorrhage; DIC: Disseminated intravascular coagulation; HELLP: Hemolysis, elevated liver enzymes, and low platelets; eGFR: Estimated glomerular filtration rate; CKD: Chronic kidney disease.

Outcome/Intervention	Frequency (%)/Mean ± SD/Median (IQR)
ICU admission	32 (26.7)
Vasopressors required	24 (20.0)
Mechanical ventilation	19 (15.8)
Blood product transfusion (any)	58 (48.3)
Disseminated intravascular coagulation	12 (10.0)
Magnesium sulfate given (HDP/eclampsia)	27 (22.5)
Antihypertensives (any)	48 (40.0)
Renal replacement therapy (any)	55 (45.8)
Intermittent hemodialysis*	41 (74.5%)
Sustained low-efficiency dialysis (SLED)	10 (18.2%)
Continuous RRT	2 (3.6%)
Peritoneal dialysis	2 (3.6%)
Number of dialysis sessions	4 (2–9)
Time to nephrology consult (hours)	10 (6–18)
Dialysis indicated at any time	55 (45.8)
Time to dialysis from admission (hours)*	24 (12–48)
Dialysis-related complications (any)*	19 (34.5%)
Intradialytic hypotension	16 (29.1%)
Catheter infection	6 (10.9%)
Bleeding	3 (5.5%)
*Obstetric complications*	
PPH	19 (15.8)
Abruption	6 (5.0)
Surgical hemostasis for PPH	9 (7.5)
*Mode of delivery (n = 112)*	
Vaginal	50 (44.6)
Cesarean	42 (37.5)
Instrumental	6 (5.4)
Pregnancy termination (medical/surgical)	8 (6.6%)
*Renal recovery at discharge*	
Complete	65 (54.2)
Partial	18 (15.0)
Dialysis-dependent	10 (8.3)
*Complications*	
Pulmonary edema	12 (10.0)
DIC	8 (6.7)
Stroke	3 (2.5)
Maternal mortality	25 (20.8)
Sepsis	12 (48.0)
Hemorrhage	6 (24.0)
Eclampsia-HELLP	5 (20.0)
Others	2 (8.0)
ICU length of stay (days)	4 (2–8)
Length of hospital stay (days)	9 (6–14)
30-day readmission	7 (5.8)
*Renal status at 6–12 weeks (among 95 survivors with follow-up)*	
eGFR ≥ 60 mL/min/1.73 m²	72 (75.7)
CKD stage ≥ 3	13 (13.7)
Dialysis-dependent	10 (10.6)

The mean gestational age at delivery was 35.4 ± 3.8 weeks. Preterm birth occurred in 56 (50.0%) women, and labor was induced in 44 (39.3%). Low birth weight (<2500 g) was observed in 63 (56.3%) neonates, with 38 (33.9%) small for gestational age; mean birth weight was 2288 ± 645 g. Apgar scores < 7 were noted in 31 (27.7%) and 22 (19.6%) at one and five minutes, respectively, with 24 (21.4%) requiring resuscitation. NICU admission was needed for 38 (34.0%) newborns, with a median stay of five days (IQR 3-9). Major neonatal complications included respiratory distress syndrome in 20 (17.9%), sepsis in 14 (12.5%), and hypoxic-ischemic encephalopathy and meconium aspiration in eight (7.1%) patients. Phototherapy was required in 26 (23.2%), and major congenital anomalies occurred in three (2.7%). Stillbirths were recorded in 22 (19.6%) and early neonatal deaths in 14 (12.5%) patients, yielding a perinatal mortality rate of 36 (32.1%), as shown in Table 5.

**Table 5:** Neonatal and perinatal outcomes among women with pregnancy-related acute kidney injury (n = 112 deliveries, unless otherwise specified) NICU: Neonatal intensive care unit; RDS: Respiratory distress syndrome; HIE: Hypoxic-ischemic encephalopathy; IUFD: Intrauterine fetal demise.

Outcome	Frequency (%)/Mean ± SD/Median (IQR)
Gestational age at delivery (weeks)	35.4 ± 3.8
Induction of labor performed	44 (39.3)
Birth weight (g), mean ± SD	2287.9 ± 645.3
Low birth weight < 2500 g	63 (56.3)
Small for gestational age (<10th centile)	38 (33.9)
Preterm birth (<37 weeks)	56 (50.0)
*Gender*	
Male	58 (51.8)
Female	54 (48.2)
*Apgar score < 7*	
At 1 minute	31 (27.7)
At 5 minutes	22 (19.6)
Resuscitation at birth required	24 (21.4)
NICU admission	38 (34.0)
NICU length of stay (days)	5 (3–9)
Meconium aspiration	8 (7.1)
*Neonatal complications*	
RDS	20 (17.9)
Sepsis	14 (12.5)
*HIE*	
Phototherapy	26 (23.2)
Congenital anomalies (major)	3 (2.7)
Stillbirth/IUFD	22 (19.6)
Early neonatal death (≤7 days)	14 (12.5)
Perinatal mortality (stillbirth + early neonatal death)	36 (32.1%)

On multivariable logistic regression analysis, lack of antenatal care registration, delayed hospital presentation beyond 48 hours, advanced renal dysfunction (KDIGO stage 3), late occurrence of AKI (third trimester or postpartum), serum creatinine >5 mg/dL, thrombocytopenia (<100 × 10^9^/L), and requirement of renal replacement therapy were identified as independent predictors of adverse maternal outcomes (aOR range: 1.7-3.4; p < 0.05). For perinatal outcomes, analysis restricted to women with completed deliveries (n = 112) demonstrated that unbooked antenatal status, delayed presentation, late-onset AKI, advanced AKI (KDIGO stage 3), elevated serum creatinine, thrombocytopenia, significant proteinuria (≥2+), and dialysis requirement were independently associated with adverse neonatal outcomes (aOR range: 1.6-2.2; p < 0.05). Maternal age ≥ 30 years showed an association with adverse perinatal outcomes on unadjusted analysis (aOR: 1.5, 95% CI: 1.0-2.5) (Table 6).

**Table 6:** Multivariable logistic regression analysis identifying independent predictors of adverse maternal (n = 120) and perinatal outcomes (n = 112 deliveries) among women with pregnancy-related acute kidney injury Logistic regression was performed using variables with p < 0.10 on univariate analysis. Results are presented as adjusted odds ratios (aOR) with 95% confidence intervals (CI). Wald χ² statistics are reported. ICU admission was considered part of the composite adverse maternal outcome and, therefore, was not included as an independent predictor. Perinatal outcome analysis was restricted to women with completed deliveries (n = 112). ^‡^Adverse maternal outcome: composite of maternal death, dialysis dependence, persistent renal dysfunction, or ICU admission. ^§^Adverse perinatal outcome: composite of stillbirth, early neonatal death, preterm birth, or NICU admission. AKI: Acute kidney injury; KDIGO: Kidney disease: improving global outcomes.

Predictor	Adverse Maternal Outcome^‡^			Adverse Perinatal Outcome^§^		
	Wald χ²	p	aOR (95% CI)	Wald χ²	p	aOR (95% CI)
ANC non-registration	5.3	0.021	1.9 (1.1–3.5)	6	0.014	2.1 (1.2–3.8)
Time to hospital > 48 h	11.4	<0.001	2.6 (1.4–4.9)	4.2	0.041	1.8 (1.0–3.3)
Timing of AKI (3rd trimester/postpartum vs ≤2nd trimester)	6.8	0.009	2.2 (1.2–4.0)	5.6	0.018	2.0 (1.1–3.7)
KDIGO stage 3	13.1	<0.001	3.4 (1.9–6.2)	4.8	0.029	2.2 (1.1–4.2)
Etiology: Sepsis (vs hypertensive)	5.7	0.017	2.5 (1.3–4.9)	3	0.082	1.5 (0.9–2.8)
Serum creatinine > 5 mg/dL	4.6	0.033	1.8 (1.0–3.2)	4.1	0.042	1.7 (1.0–3.0)
Platelets < 100 ×10⁹/L	4.2	0.042	1.7 (1.0–3.0)	5.3	0.021	2.0 (1.1–3.6)
Proteinuria ≥ 2+	2.9	0.09	1.4 (0.8–2.6)	4.3	0.038	1.6 (1.1–2.9)
Dialysis required	12	<0.001	3.1 (1.7–5.6)	5.8	0.016	1.9 (1.1–3.4)
Age ≥ 30 years (unadjusted)	3	0.082	—	4.1	0.043	1.5 (1.0–2.5)

## Discussion

Our prospective study of 120 women with pregnancy-related acute kidney injury (PR-AKI) highlights the persistent burden of this condition in low-resource settings, despite advances in obstetric and nephrological care [6,7,9]. The majority of affected women were young (<30 years), rural residents with poor antenatal coverage, and many presented late after symptom onset. These sociodemographic determinants mirror the observation by Meena et al. that nearly half of women with PR-AKI receive no antenatal care, predisposing them to delayed diagnosis and preventable complications (pooled coverage ~49%) [13].

Hypertensive disorders of pregnancy, including preeclampsia and eclampsia, were the most common cause in our cohort (37.5%), followed by obstetric hemorrhage and sepsis. This pattern is consistent with global pooled estimates by Trakarnvanich et al., showing preeclampsia accounts for 36.6% of PR-AKI cases [14]. In South Asia, studies by Meena et al. and Berhe et al. showed that preeclampsia contributes 33%-44% of cases, while sepsis (including puerperal infections) and hemorrhage together exceed 50% [13,15]. Regional studies underscore similar distributions: in a 10-year South Indian study by Sahay et al., preeclampsia (44%), sepsis (33%), and hemorrhage (19%) were the leading causes [16]; while in Kashmir, septic abortion and anemia predominated [17]. These findings collectively suggest that preventable obstetric complications remain a key driver of PR-AKI in India, in contrast to high-income settings where chronic hypertension, preeclampsia, and preexisting renal disease predominate [18].

In our study, nearly half (45.8%) of patients required renal replacement therapy, which falls within the global range where 37% (95% CI: 26-50%) of PR-AKI patients need dialysis [19]. In India, studies by Banerjee and Mehrotra as well as Sandilya et al. report even higher dialysis rates (60%-73%) [20,21]. The favorable finding in our study was that more than half of women regained normal renal function at discharge, though 8% remained dialysis-dependent, echoing the global burden of persistent renal dysfunction (8.5%) [19]. Similarly, South Indian cohorts have reported 8%-15% progression to ESRD [16]. Histopathologic studies by Kumar et al. and Kumari et al. indicate that renal cortical necrosis, often associated with sepsis and hemorrhage, accounts for up to 43% of severe PR-AKI cases [22,23], explaining the irreversible renal impairment observed in a subset of women.

Maternal mortality in our cohort was 21%, which, although alarming, is consistent with other Indian reports where mortality ranged between 5% and 21% [20,21]. Meta-analytic evidence shows PR-AKI increases maternal mortality 4.5-fold (OR: 4.50; 95% CI: 2.73-7.43) with a pooled mortality of 12.7% [15]. Predictors of poor maternal outcome in our study included delayed presentation, advanced KDIGO stage, high serum creatinine, thrombocytopenia, dialysis requirement, and ICU admission, factors widely corroborated in prior Indian series [24,25]. Together, these reinforce the critical importance of early diagnosis, timely referral, and multidisciplinary management.

The perinatal toll was equally concerning. Half of the neonates were preterm, 56% had low birth weight, one-third required NICU admission, and overall perinatal mortality reached 32%. This aligns with South Asian data showing fetal mortality rates of 30%-48% in PR-AKI [13,25]. Global evidence confirms a 3.4-fold higher risk of stillbirth or perinatal death (OR: 3.39; 95% CI: 2.76-4.18) [14]. Additionally, PR-AKI pregnancies tend to end earlier (-0.7 weeks of gestation), and neonates weigh significantly less (-740 g) [26]. These findings reflect the dual impact of maternal hemodynamic instability and placental dysfunction, leading to intrauterine growth restriction, prematurity, and neonatal complications [24].

Contrastingly, studies from developed nations report much lower maternal mortality (<5%) and better neonatal survival, attributable to robust antenatal coverage, early recognition of hypertensive disorders, and access to critical care and dialysis facilities [24]. The disparity underscores the need to address systemic healthcare gaps in India, including strengthening rural obstetric care, universal antenatal screening, and streamlined referral pathways.

Implications

The findings underscore PR-AKI as a sentinel indicator of gaps across the continuum of obstetric and nephrology care. Programmatic priorities should include strengthening antenatal care coverage, earlier identification and management of hypertensive disorders, robust infection prevention and sepsis control, and timely access to blood products. Given the adverse outcomes associated with late presentation and advanced AKI, streamlined referral pathways and integrated obstetric-nephrology care models are essential. Regionally coordinated dialysis support and standardized care bundles may further reduce maternal and perinatal morbidity and mortality.

Strengths and limitations

Key strengths of this study include its prospective design, standardized application of KDIGO criteria, and comprehensive assessment of maternal and perinatal outcomes with multivariable analyses. Several limitations merit consideration. Being a single-center study, generalizability may be limited. The postpartum subgroup was smaller than the antenatal cohort, which necessitates cautious interpretation of postpartum-specific etiologies. Perinatal analyses were restricted to completed deliveries, and long-term maternal renal outcomes and child neurodevelopment could not be evaluated due to limited follow-up. As an observational study, associations rather than causal inferences can be drawn. Future multicenter studies with longer follow-up are needed to validate these findings and examine long-term outcomes.

## Conclusions

PR-AKI remains a substantial and largely preventable contributor to maternal and perinatal morbidity and mortality in India. The predominance of hypertensive disorders, sepsis, and obstetric hemorrhage reflects ongoing gaps in antenatal surveillance, timely recognition, and referral. Adverse outcomes are more frequent with delayed presentation and advanced AKI, emphasizing the importance of early detection and coordinated care. Strengthening antenatal services, improving referral systems, and fostering multidisciplinary management involving obstetricians, nephrologists, and critical care teams are pivotal to improving survival and renal recovery for mothers and optimizing neonatal outcomes.

## References

[1] Cheung KL, Lafayette RA (2013). Renal physiology of pregnancy. Adv Chronic Kidney Dis.

[2] Beers K, Patel N (2020). Kidney physiology in pregnancy. Adv Chronic Kidney Dis.

[3] Prakash J, Ganiger VC (2017). Acute kidney injury in pregnancy-specific disorders. Indian J Nephrol.

[4] Rao S, Jim B (2018). Acute kidney injury in pregnancy: the changing landscape for the 21st century. Kidney Int Rep.

[5] Reddy KH, Kumar KP, Rajan K, Shaik S (2015). Pregnancy related acute kidney injury: nondialytic management. Int J Reprod Contracept Obstet Gynecol.

[6] Prakash J, Ganiger VC, Prakash S, Iqbal M, Kar DP, Singh U, Verma A (2018). Acute kidney injury in pregnancy with special reference to pregnancy-specific disorders: a hospital based study (2014-2016). J Nephrol.

[7] Prakash J, Prakash S, Ganiger VC (2019). Changing epidemiology of acute kidney injury in pregnancy: a journey of four decades from a developing country. Saudi J Kidney Dis Transpl.

[8] Szczepanski J, Griffin A, Novotny S, Wallace K (2020). Acute kidney injury in pregnancies complicated with preeclampsia or HELLP syndrome. Front Med (Lausanne).

[9] Gautam M, Saxena S, Saran S, Ahmed A, Pandey A, Mishra P, Azim A (2022). Etiology of pregnancy-related acute kidney injury among obstetric patients in India: a systematic review. Indian J Crit Care Med.

[10] İnan C, Uygur L, Alpay V (2024). Hypertensive disorders of pregnancy: diagnosis, management and timing of birth. Balkan Med J.

[11] Sachan R, Shukla S, Shyam R, Sachan PL, Patel ML (2022). Feto-maternal outcome of pregnancy related acute kidney injury in a North Indian population. J Family Community Med.

[12] Kellum JA (2015). Diagnostic criteria for acute kidney injury: present and future. Crit Care Clin.

[13] Meena P, Das P, Auradkar A (2025). Pregnancy-associated acute kidney injury as an important driver of chronic kidney disease in females in developing countries: a systematic review. Matern Health Neonatol Perinatol.

[14] Trakarnvanich T, Ngamvichchukorn T, Susantitaphong P (2022). Incidence of acute kidney injury during pregnancy and its prognostic value for adverse clinical outcomes: a systematic review and meta-analysis. Medicine (Baltimore).

[15] Berhe E, Teka H, Abraha HE (2024). Characteristics and outcome of pregnancy-related acute kidney injury in a teaching hospital in a low-resource setting: a five-year retrospective review. BMC Nephrol.

[16] Sahay M, Priyashree Priyashree, Dogra L, Ismal K, Vali S (2022). Pregnancy-related acute kidney injury in public hospital in South India: changing trends. J Assoc Physicians India.

[17] Najar MS, Shah AR, Wani IA, Reshi AR, Banday KA, Bhat MA, Saldanha CL (2008). Pregnancy related acute kidney injury: a single center experience from the Kashmir Valley. Indian J Nephrol.

[18] Godara SM, Kute VB, Trivedi HL (2014). Clinical profile and outcome of acute kidney injury related to pregnancy in developing countries: a single-center study from India. Saudi J Kidney Dis Transpl.

[19] Soto ASM, Piña MDR, Benhumea AMS, Zerón HM (2024). Use of renal replacement therapy in pregnant women with acute kidney injury or chronic kidney disease: a systematic review. Acta Med Philipp.

[20] Banerjee A, Mehrotra G (2020). Comparison of standard conservative treatment and early initiation of renal replacement therapy in pregnancy-related acute kidney injury: a single-center prospective study. Indian J Crit Care Med.

[21] Sandilya S, Rani KU, Kumar R (2023). Risk factors and fetomaternal outcome in pregnancy-related acute kidney injury. J Family Med Prim Care.

[22] Kumar A, Rajput M, Kumar R (2025). Postpartum renal cortical necrosis: experience from a tertiary care center in India. J Nephrol.

[23] Kumari P, Trivedi K, Banerjee S, Sharma A, Sinha T, Boipai P, Kumari S (2025). Study on incidence of pregnancy-related acute kidney injury and its associated risk factors and outcomes: in preponderant tribal state of India. Ann Afr Med.

[24] Davidson B, Bajpai D, Shah S (2022). Pregnancy-associated acute kidney injury in low-resource settings: progress over the last decade. Semin Nephrol.

[25] Mahalingam M, Sundarraj MT, Mohan G (2024). Pregnancy-related acute kidney injury: our experience in a tertiary care center. J South Asian Feder Obst Gynae.

[26] Cooke WR, Hemmilä UK, Craik AL (2018). Incidence, aetiology and outcomes of obstetric-related acute kidney injury in Malawi: a prospective observational study. BMC Nephrol.

